# Unravelling the Cu and Ce Effects in MnO_2_-Based Catalysts for Low-Temperature CO Oxidation

**DOI:** 10.3390/nano15030166

**Published:** 2025-01-22

**Authors:** Egor D. Blinov, Ekaterina V. Kulchakovskaya, Nikolai A. Sokovikov, Valery A. Svetlichnyi, Sergei A. Kulinich, Olga V. Vodyankina

**Affiliations:** 1 Department of Physical and Colloid Chemistry, Faculty of Chemistry, Tomsk State University, Tomsk 634050, Russia; 2 Department of Physical Chemistry, Faculty of Natural Science, Novosibirsk State University, Novosibirsk 630090, Russia; 3 Laboratory of Advanced Materials and Technology, Tomsk State University, Tomsk 634050, Russia; 4 Research Institute of Science and Technology, Tokai University, Hiratsuka 259-1292, Kanagawa, Japan

**Keywords:** Cu- and/or Ce-modified MnO_2_ catalyst, nanodispersed active species, OMS-2, cryptomelane, pyrolusite, TPR-H_2_, Raman, low-temperature CO oxidation

## Abstract

Cu-containing and Ce-modified OMS-2 catalysts were prepared at various calcination temperatures using the hydrothermal method and tested for low-temperature CO oxidation. The structure, chemical compositions, and physical–chemical properties of the catalysts were characterized using XRD, N_2_ physisorption, XRF, Raman spectroscopy, SEM, high-resolution TEM with EDX, TPR-H_2_, and XPS. The incorporation of Cu into the Ce-OMS-2 sample facilitated the transformation of pyrolusite into cryptomelane, as confirmed by Raman spectroscopy data. In the light-off mode, the Cu/Ce-OMS-2-300 and Cu/OMS-2 samples exhibited higher activity in low-temperature CO oxidation (T_90_ = 115 and 121 °C, respectively) compared to sample Cu/Ce-OMS-2-450. After a long-run stability test, the Cu/Ce-OMS-X samples demonstrated excellent performance: the T_80_ increased by 16% and 7% for the samples calcined at 300 °C and 450 °C, respectively, while the T_80_ for the Cu/OMS-2 increased by 40%. The Cu/OMS-2 and Cu/Ce-OMS-2-300 samples were found to have an increased content of nanodispersed copper sites on their surfaces. These copper sites contributed to the formation of the Cu^2+^-O-Mn^4+^ interface, which is responsible for the CO oxidation. The presence of Ce^3+^ in the catalyst was found to increase its stability in the presence of water vapor due to the higher reoxidation ability in comparison with Ce-free sample Cu/OMS-2.

## 1. Introduction

Nowadays, the problem of environmental pollution is topical. When fossil fuels actively used in industries (for vehicles, power plants, etc.) are burned, large amounts of pollutants such as volatile organic compounds (VOCs), CO, NO_x_, soot particles, etc., are released into the air, which can cause serious harm to nature and human health [1,2,3]. One of the most common ways to purify the air from CO is its low-temperature catalytic oxidation [4].

The catalytic oxidation of CO has been studied for a long time, and many catalysts have been proposed for this process. However, most of them are based on noble metals that dramatically increase the final price of the catalysts [5,6,7,8,9]. In this regard, in recent years, there has been a search for alternative catalysts for CO oxidation using less expensive transition metals [5,10,11,12,13]. Promising catalysts in this area are systems based on MnO_2_ with a cryptomelane structure (OMS-2). This compound is a 2 × 2 tunnel octahedral molecular sieve structure built by connecting the edges and common angles of MnO_6_ clusters with an edge length of 4.6 Å [14]. The advantages of the OMS-2 include the mobility of lattice oxygen and the mixed valence of Mn, which allow it to be used as a catalyst for oxidation/decomposition processes. For instance, the use of OMS-2 was reported for the processes of the decomposition of ozone [15,16,17] and organic compounds [18,19,20], the oxidation of VOCs and CO, NO_x_ reduction [5,21,22,23,24], etc. The approach to modify the OMS-2 with cations of various metals (such as Ce, Cu, Co, Fe, Cr, etc. [15,20,24]) is often used to change its characteristics and improve catalytic properties. The introduced metal cations can either be built into the tunnels replacing potassium ions or built directly into the OMS-2 framework, replacing manganese ions.

The Ce-containing OMS-2 is one of the most promising catalysts [15,16,20,25,26]. However, the Ce addition changes the structure and morphology of the OMS-2, which is accompanied by the appearance of new CeMnO_x_ structures [16,27], while due to the redox transition between Ce^3+^ ↔ Ce^4+^, such systems exhibit an enhanced oxygen storage capacity [16]. By now, a large number of methods to prepare the Ce-OMS-2 have been proposed, namely, hydrothermal synthesis [15,28], the sol–gel method [26], the reflux approach [29,30], as well as ion exchange [31,32,33,34]. It is also known from the literature that one of the most effective base metals for the CO oxidation process is copper, since Cu^+^ ions promote easy CO oxidation in the CuO-CeO_2_ system [35,36]. In this case, Cu cations should be formed on the surface as a result of the partial reduction in CuO_x_ and be accessible to CO molecules [37,38]. Several studies [35,39,40] showed that the oxidation-reduction reaction Ce3^+^ + Cu^2+^ ↔ Ce^4+^ + Cu^+^ can occur in the CuCeO_x_ catalysts. This interaction causes the synergistic effect of Cu-Ce mixed oxide systems in comparison with the individual CuO and CeO_2_ [39].

Thus, the behavior of catalytic systems based on OMS-2 in the simultaneous presence of Ce^n+^ and Cu^m+^ cations and their mutual influence on the CO oxidation process was not studied. In the present work, the modified Ce- and Cu,Ce-containing OMS-2 catalysts were synthesized and studied in comparison with the Cu/OMS-2 to unravel the Cu and Ce effects in the MnO_2_-based catalysts for low-temperature CO oxidation.

## 2. Experimental Section

### 2.1. Preparation of Materials

The OMS-2 samples were prepared by hydrothermal synthesis according to the procedure described in Ref. [27]. Copper was introduced into the OMS-2 and Ce–OMS-2 samples in an amount of 4 wt.% by the incipient wetness impregnation method. The Cu(NO_3_)_2_·3H_2_O (analytical grade) was used as a copper precursor. The prepared samples of copper-containing catalysts were dried at 120 °C for 12 h, then calcined at 300 °C (Cu/Ce–OMS-2-300) or 450 °C (Cu/Ce–OMS-2-450 and Cu/OMS-2) for 2 h.

### 2.2. Characterization of Materials

X-ray phase analysis was carried out by the sliding beam method on the XRD-7000 diffractometer (Shimadzu, Kyoto, Japan) using CuKα radiation (λ = 1.5418 Å) and a K_β_ filter. Recording conditions were as follows: scanning rate was 2 deg/min within a range from 10 to 80 deg, radiation intensity was 1000 units, tube voltage was 30 kV, and current was 30 mA. Phase composition was analyzed using the PDF-4 database (Release 2022). To refine the crystal lattice parameters and determine the coherent scattering regions (CSR) for crystalline phases, the POWDER CELL 2.4 full-profile analysis program (Werner Kraus & Gert Nolze, Berlin, Germany) was used.

The elemental compositions of the supports and catalysts were determined using the XRF-1800 sequential wave-dispersive X-ray fluorescence spectrometer (Shimadzu, Kyoto, Japan).

The Raman spectra of the catalysts under study were obtained using the InVia Basic spectrometer (Renishaw, Wotton-under-Edge, UK) with the Leica DM2500 M microscope (Leica, Wetzlar, Germany). The samples were excited by a laser with a wavelength of 785 nm.

The characteristics of the porous structure of the samples were measured using low-temperature nitrogen adsorption–desorption data at −196 °C using the 3Flex automatic gas adsorption analyzer (Micrometritics, Norcross, GA, USA). The specific surface area (S_BET_) was determined using the multi-point (10–12 points) Brunauer–Emmett–Teller (BET) method in the range of relative nitrogen pressures P/P_0_ from 0.05 to 0.30. Before measuring the specific surface area, the samples were degassed (150 °C, 2 h, vacuum).

The reactivity of the samples was studied by the temperature-programmed reduction with hydrogen (TPR-H_2_) on the AutoChem HP 2950 chemisorption analyzer (Micromeritics, Norcross, GA, USA). The reduction was carried out in the temperature range of 25–700 °C in a hydrogen flow (10% vol. in Ar) at a flow rate of 20 mL/min and a heating rate of 10 °/min. The samples were subjected to oxidative pretreatment in an air flow at 450 °C.

The X-ray photoelectron spectra (XPS) were recorded using the KRATOS ES-300 analytical spectrometer (Kratos Analytical, Manchester, UK). Before the measurements, the samples were ground and applied to double-sided carbon tape. The Kα line of magnesium with an energy of hv = 1253.6 eV was used as the primary radiation. The power of the X-ray source was 80 W in all cases. The internal calibration of the spectra was carried out by setting the position of the maximum of the C 1s peak to 284.8 eV.

Morphology was studied by scanning electron microscopy (SEM) using Vega 3H Tescan microscope (Tescan, Brno, Czech Republic) and transmission electron microscopy (TEM) using Themis Z microscope (Thermo Fisher Scientific, Waltham, MA, USA) with a two-corrector astigmatism regulation system at an accelerating voltage of 200 kV. Local elemental analysis was carried out according to the energy-dispersive X-ray spectroscopy (EDX) data using the Super-X EDX spectrometer (Thermo Fisher Scientific, Waltham, MA, USA). Dark-field images were obtained in the scanning (STEM) mode using the HAADF (High-Angle Annular Dark-Field) detector to record electrons scattered at large angles.

### 2.3. Catalytic Tests

The catalytic properties of the samples were studied in the CO oxidation reaction using a catalytic unit (Katacon LLC, Novosibirsk, Russia) equipped with a flow U-shaped fixed-bed reactor and online chromatographic analysis. The experimental procedure included sample pretreatment followed by testing at a given reaction temperature. For the experiment, 0.25 cm^3^ of a sample with a particle size of 0.25–0.50 mm diluted with 0.25 cm^3^ of quartz with a particle size of 0.25–0.50 mm was used. The pretreatment of the samples was carried out in a flow of a mixture of 21% O_2_ in He (25 mL/min) by heating the sample to the required temperature, then holding for 20 min and cooling down to 50 °C. Then, the flow was switched to the reaction mixture, the reactor was heated in 10–20 °C increments to the required reaction temperature until 100% conversion was achieved, and then the temperature was reduced in 10–20 °C increments to obtain the light-off curves. The reaction mixture was an oxygen-depleted mixture of the following composition: 1 vol.% CO, 8 vol.% O_2_.

For the stability experiments, after switching to the reaction mixture, the reactor was also heated stepwise to the required temperature (initial CO conversion of no less than 80%), and catalytic tests were carried out at a given CO conversion for 25 h. The initial reaction mixture simulated real exhaust conditions and contained 1 vol.% CO, 21 vol.% O_2_ and 10 vol.% H_2_O in helium. The flow rate for all experiments was 375 mL·min^−1^.

## 3. Results and Discussion

### 3.1. Composition and Structure of Catalysts

Table 1 shows the elemental composition and textural characteristics of the prepared catalysts, and Figure S1 exhibits the textural characteristics of the prepared catalysts, including their adsorption–desorption isotherms and pore size distribution. The original unmodified OMS-2 sample is characterized by a specific surface area of 15.9 m^2^/g and a total pore volume of 0.07 cm^3^/g. The Cu introduction by incipient wetness impregnation from a copper nitrate solution onto the unmodified OMS-2 (Cu/OMS-2 sample) has a slight effect on the pore characteristics of the catalyst; the specific surface area and pore volume values remain virtually unchanged.

The introduction of cerium nitrite at the stage of the co-precipitation of manganese salt solutions followed by hydrothermal treatment allows introducing no more than 1.6 mol.% of the modifier into the composition of the Ce–OMS-2 catalyst instead of the specified 5 mol.% (Table 1). The content of potassium cations localized in the tunnels decreases ~3 times compared to the unmodified OMS-2, which allows us to conclude both about the preferential introduction of cerium cations into the OMS-2 tunnels with the replacement of potassium ions and about the formation of a phase different from the OMS-2 structure, with subsequent leaching of the potassium ions at the stage of washing after the hydrothermal treatment.

For the cerium-modified sample, an increase in the specific surface area to 46.5 m^2^/g and the total pore volume to 0.17 m^3^/g is observed. The pore size distribution curves (Figure S1) show that, in general, for the Ce-containing samples, the contribution of mesopores with sizes of ~10 nm increases compared to the unmodified OMS-2. The nature of the mesopore size distribution curves for the modified Ce-OMS-2 is bimodal with a maximum at 2–4 nm and predominant pores in the range of 8.5–12 nm. The Cu introduction into the Ce-OMS-2 by incipient wetness impregnation leads to a slight decrease in the specific surface area.

The influence of the added Cu and/or Ce components can be observed from the results of morphological studies in Figure S2. The appearance of dispersed particles along with the rod-shaped one’s characteristic of the cryptomelane phase is detected in the Ce-OMS-2. The introduction of the Cu-containing component does not change the morphology of the obtained samples.

**Figure 1:**
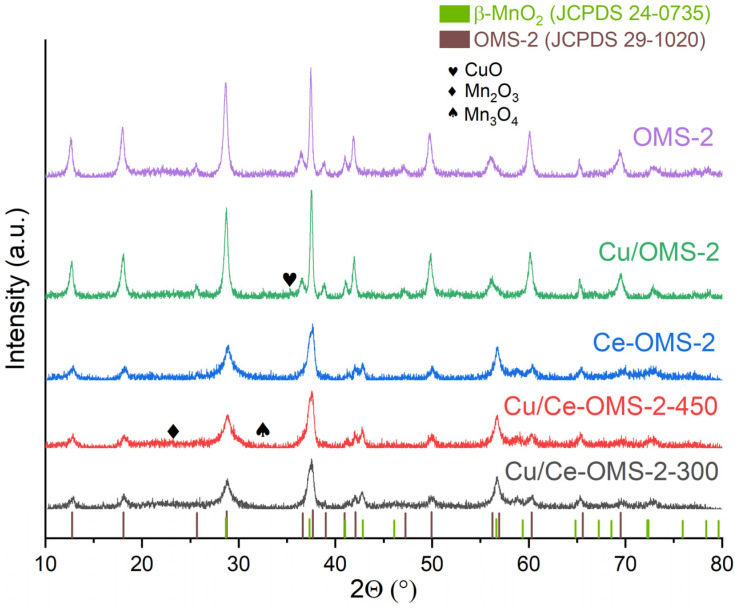
XRD patterns for prepared catalysts.

Figure 1 shows the diffraction patterns for the obtained samples. The main reflections correspond to the MnO_2_ phases, except for the Cu/OMS-2 sample, for which a low-intensity reflection at 35.5° is detected, which can be attributed to the monoclinic CuO phase [22]. The Ce-modified samples consist of two phases: cryptomelane (JCPDS No. 29-1020) and pyrolusite (JCPDS No. 24-0735). In Figure S3, the unit cells for the abovementioned phases are presented. Low-intensity reflections at 23.11° and 32.46° in the pattern for the Cu/Ce-OMS-2-450 sample appear. These can be attributed to the Mn_2_O_3_ (JCPDS No. 089-4836) and Mn_3_O_4_ (JCPDS No. 024-073) phases, respectively. Table 2 represents the results of the XRD analysis of the investigated samples. Figures S4–S7 show the detailed results of the sample structure modeling in PDF 4+.

The cerium introduction at the stage of MnO_x_ precursor co-precipitation before the hydrothermal treatment in the OMS-2 prevents the formation of the 2 × 2 OMS-2 tunnels. Two mechanisms were proposed in the literature that describe the growth of the anisotropic tunnel structures of MnO_2_. For these mechanisms, the intermediate compound is the layered structure of δ-MnO_2_. As it was shown in Ref. [41], the first mechanism describes the partial dissolution of δ-MnO_2_ layers with subsequent recrystallization into α-MnO_2_. However, this mechanism does not describe the possible formation of the pyrolusite phase during the hydrothermal treatment. On the other hand, the second mechanism describes the rolling of the layers of the δ-MnO_2_ structure with the possible formation of either α-MnO_2_ or β-MnO_2_ [42]. In this case, an important factor in the formation of α-MnO_2_ or β-MnO_2_ is the presence of a stabilizing cation, such as K^+^, in an optimal amount. In the case of the introduction of Ce(NO_3_)_3_ ions before the hydrothermal treatment procedure, the adsorption of Ce^3+^ ions on the surface of δ-MnO_2_ layers causes the electrostatic repulsion of K^+^. This effect causes the destruction of metastable 2 × 2 tunnels to form 1 × 1 tunnels.

Thus, the appearance of two phases in the composition of the synthesized samples is associated with the influence of Ce^3+^ cations introduced at the stage of the co-precipitation of the catalyst precursor before the hydrothermal treatment. Copper is distributed in the form of highly dispersed sites on all Ce-OMS-2 samples. For the Cu/OMS-2, the appearance of a reflection of the CuO phase is detected, which indicates a decrease in the dispersion of the Cu-containing species on the catalyst surface.

**Figure 2:**
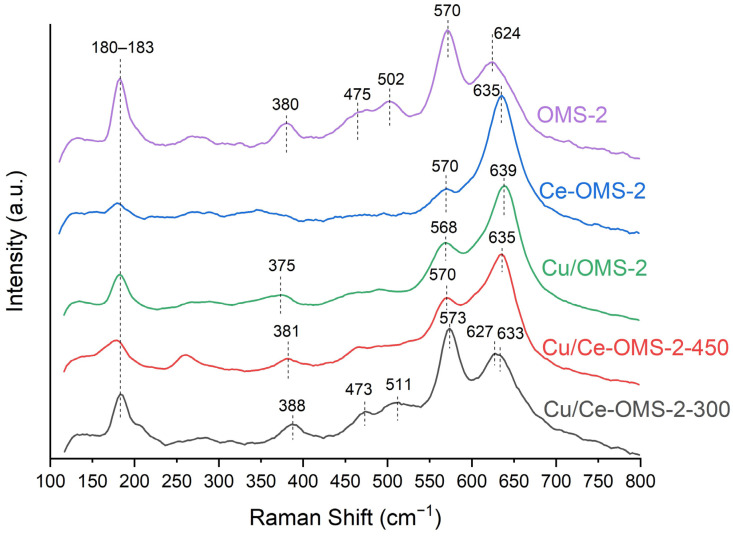
Raman shift spectra of prepared catalysts.

The Raman spectrum for the unmodified OMS-2 sample (Figure 2) is consistent with the one shown in Refs. [43,44]. The main bands characterizing the OMS-2 structure are those in the region of 570 and 630 cm^−1^. The band at a frequency of 570–572 cm^−1^ corresponds to the displacement of oxygen atoms relative to the manganese ones along the octahedral chains of the OMS-2 rods. The band in the region of 630 cm^−1^ characterizes the stretching vibrations of the manganese–oxygen bond perpendicular to the direction of the chains. The presence of heavy cations in the tunnels of the structure usually reduces the vibrational Mn-O components, which are vertical to the octahedral chains and protrude into the 2 × 2 tunnels, which can be characterized by a decrease in the intensity of this band. In addition, the band at ~386 cm^−1^ can be considered characteristic and can be described as the deformation vibrations of the manganese bond with oxygen. A band in the region of 180 cm^−1^ was attributed to the flexing of the overall octahedral framework with a nanorod structure [45].

The copper introduction by the incipient wetness impregnation method in the OMS-2 sample leads to some changes in the OMS-2 structure (Figure 2). A shift in the band at 621 cm^−1^ toward higher wavenumbers up to 640 cm^−1^ as well as a noticeable decrease in intensity and a slight shift in the band at 570 cm^−1^ are observed. These data may indicate both the formation of Mn^4+^-O-Cu^2+^ bonds on the surface of anisotropic particles and the Mn^4+^ substitution with copper ions in the MnO_6_ octahedra in the OMS-2 framework, as shown previously in Ref. [46]. Such a substitution reduces the intensity of the 570 cm^−1^ band due to a decrease in the number of MnO_6_ octahedra in the overall structure as well as the one of the band at 180 cm^−1^ due to a change in the nature of bonds in the framework. The shift in the band at 624 cm^−1^ toward higher wavenumbers also confirms the incorporation of copper cations into the framework. Copper has a higher Pauling electronegativity than manganese, which can cause a shift in the electron density from manganese to copper and, as a result, a weakening of the Mn-O bond.

The Ce introduction at the stage of precursor co-precipitation before the hydrothermal treatment during the synthesis of the Ce-OMS-2 sample changes the appearance of the Raman spectrum to an even greater extent, and along with the shift in the band at 635 cm^−1^, an even greater decrease in the intensity of the one at 180 cm^−1^ is also detected. The increase in intensity and the shift in the band at 624 cm^−1^ toward higher wavenumbers indicate the contribution of the pyrolusite structure, the formation of which is confirmed by the XRD method. The decrease in the intensities of the bands at 570 cm^−1^ and 180 cm^−1^ is due to a decrease in the cryptomelane content in the overall composition of the Ce-OMS-2 sample, which is also confirmed by the disappearance of the band at 380 cm^−1^.

The Cu introduction into the Ce–OMS-2 sample is accompanied by changes in the Raman spectrum. The appearance of a band at 380–388 cm^−1^, responsible for the deformation vibrations of the Mn–O bond, and an increase in the intensity of the band at 180 cm^−1^ are noticeable. The band at 570–572 cm^−1^ also becomes more pronounced. Two sharp high-frequency Raman bands in the region of 570 and 630 cm^−1^ indicate a well-developed tetragonal structure with an interstitial space consisting of 2 × 2 tunnels (spectrum for Cu/Ce-OMS-2-300) [47]. It is noteworthy that the splitting of the band in the region of 630 cm^−1^ with two maxima at 627 cm^−1^ and 633 cm^−1^ was found for the Cu/Ce-OMS-2-300 sample. The first peak corresponds to the Mn-O oscillations characteristic of the cryptomelane structure, the second one is similar to oscillations characteristic of the pyrolusite structure. Thus, copper introduction in the Ce–OMS-2 sample followed by heat treatment at 300 °C allows the partial reconstruction of the cryptomelane structure compared to the Ce-OMS-2 sample structure.

According to the XRD results, an increase in the calcination temperature of the Cu/Ce-OMS-2-450 from 300 °C to 450 °C leads to an additional rearrangement of the cryptomelane structure into pyrolusite with the appearance of new Mn_2_O_3_ and Mn_3_O_4_ phases. In Ref. [48], it was shown that the pyrolusite structure with 1 × 1 tunnels can be rearranged with the formation of a cryptomelane-like structure with 2 × 2 channels due to the rotation of the corresponding octahedral units by 45°. The Cu introduction, as was mentioned earlier, can lead to the substitution of manganese ions and/or their replenishment in the corresponding cation vacancies by [CuO_6_] octahedra. The formation of such cation vacancies was predicted by the DFT calculation upon the introduction of Ce^3+^/Ce^4+^ cations into the OMS-2 channels [28]. In this case, the overall positive charge of the framework decreases due to the lower oxidation state of Cu^2+^ compared to Mn^4+^/Mn^3+^. Due to this, the structure can be rearranged into 2 × 2 tunnels, inside which, instead of K^+^, there will be cations of cerium and/or copper (similarly to the natural minerals, hollandite and coronadite, in which the 2 × 2 tunnels contain ions of Ba^2+^ and Pb^2+^, respectively [49]).

Thus, the copper introduction facilitates the reconstruction of the 2 × 2 structure on the surface of pyrolusite particles in the Cu/Ce-OMS-2-300 sample, while a temperature increase to 450 °C leads to phase separation in the structure of the Cu/Ce-OMS-2-450 sample with the appearance of Mn_2_O_3_, Mn_3_O_4_, and highly dispersed CuO particles.

### 3.2. Temperature-Programmed Reduction by H_2_

**Figure 3:**
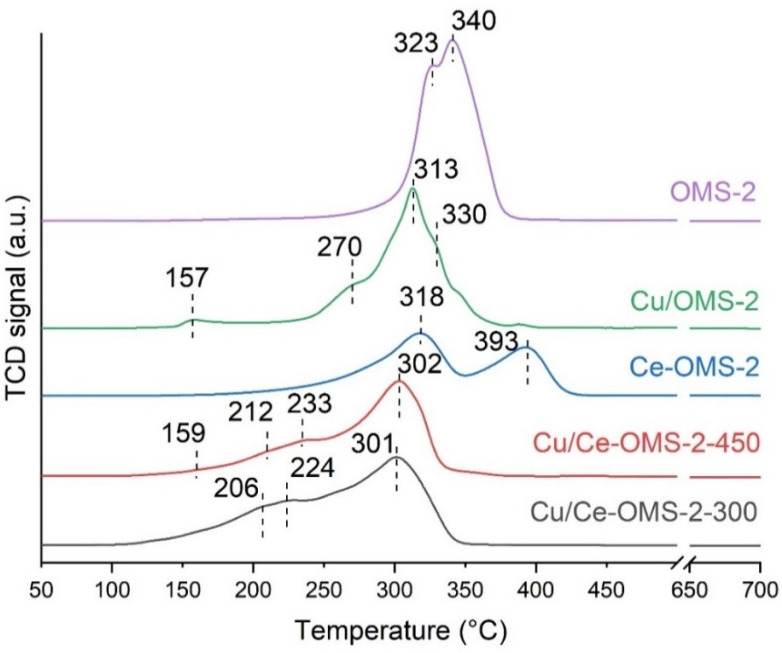
TPR-H_2_ profiles prepared catalysts.

Figure 3 shows the results of TPR-H_2_ for the studied catalyst samples. Two consumption maxima at 323 °C and 340 °C for the OMS-2 characterize two stages of the MnO_2_ reduction: the first one to Mn_2_O_3_/Mn_3_O_4_, and then a complete reduction to MnO [17]. The introduction of copper and/or cerium into the OMS-2 structure leads to significant changes in the hydrogen consumption profiles.

The copper introduction into the system is accompanied by a general shift in the TPR profile toward lower temperatures (313 °C and 330 °C for two stages of the MnO_2_ reduction) compared to unmodified OMS-2, which indicates close contact of the copper cations with MnO_2_ and the formation of Cu-O-Mn bonds. At the same time, the appearance of two new consumption bands is noticeable: the peak at 157 °C is referred to by the reduction in dispersed copper oxide on the OMS-2 surface, the second one at 270 °C is attributed to the reduction in larger CuO particles [5]. The appearance of larger CuO particles for this sample is confirmed by XRD (Figure 1).

The cerium introduction further changes the shape of the reduction profile. An additional peak of hydrogen consumption is observed at higher temperatures with a maximum at 393 °C that can be associated with the formation of pyrolusite, which features higher reduction temperatures compared to the cryptomelane structure due to higher stability [50].

After the copper introduction into the Ce-OMS-2 system, a profile that is more characteristic of the OMS-2 structure is observed compared to the Ce-OMS-2 sample. The high-temperature peak disappears, and the general appearance of the profile is more characteristic of the Cu/OMS-2 than of the Ce-OMS-2 before the copper introduction. For the Cu/Ce-OMS-2-450 sample, a low-intensity peak at 159 °C corresponding to the reduction in highly dispersed copper oxide is noticeable. Table 3 shows total H_2_ consumption for all samples.

The TPR-H_2_ profiles in the case of the Cu/Ce-OMS-2-X samples become more characteristic of the OMS-2 structure compared to the Ce-OMS-2 sample. Thus, the copper introduction facilitates the reconstruction of the cryptomelane structure with the appearance of characteristic bands in the Raman spectrum.

### 3.3. Catalytic Properties and Stability in Presence of Water Vapor

**Figure 4:**
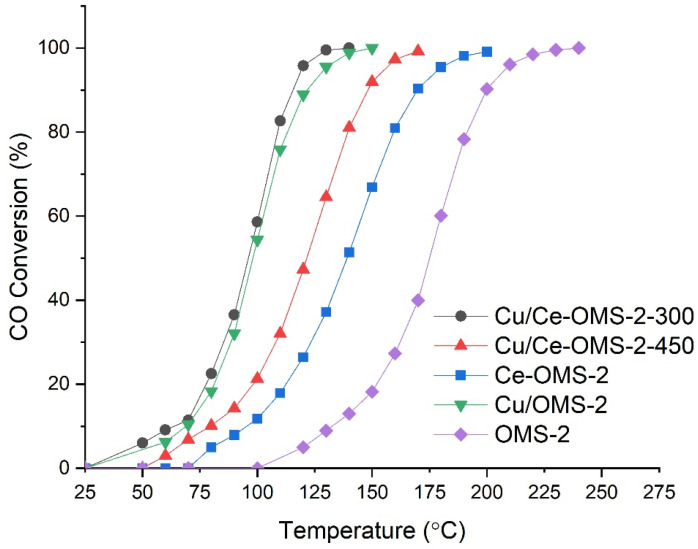
Catalytic activity of synthesized catalysts.

Figure 4 shows the catalytic data for the OMS-2 samples modified with copper and/or cerium in low-temperature CO oxidation in the light-off mode. It is evident that the introduction of both copper and cerium into the OMS-2 increases the catalytic activity in low-temperature CO oxidation. The Cu/OMS-2 sample, where copper is deposited by the incipient wetness impregnation method, increases the activity to a greater extent compared to the Ce–OMS-2. The Cu/OMS-2 sample is the most active catalyst. For this sample, the temperature to achieve 90% conversion (T_90_) is 121 °C, while those for Ce–OMS-2 and unmodified OMS-2 are 170 and 200 °C, respectively. With the copper introduction into the Ce-OMS-2, the catalytic activity is improved. Thus, for the Cu/Ce–OMS-2-300 and Cu/Ce–OMS-2-450 samples, the T_50_ values are 96 °C and 122 °C, and the T_90_ values are 115 °C and 148 °C, respectively. The Cu/OMS-2 and Cu/Ce-OMS-2-300 are the most active of the prepared catalysts in the CO oxidation process. A detailed comparison of the catalytic properties of sample Cu/Ce-OMS-2-300 with the state-of-the-art catalysts previously reported in the literature (Table S1) shows its comparable, or superior, performance even compared with its counterparts containing noble metals.

**Figure 5:**
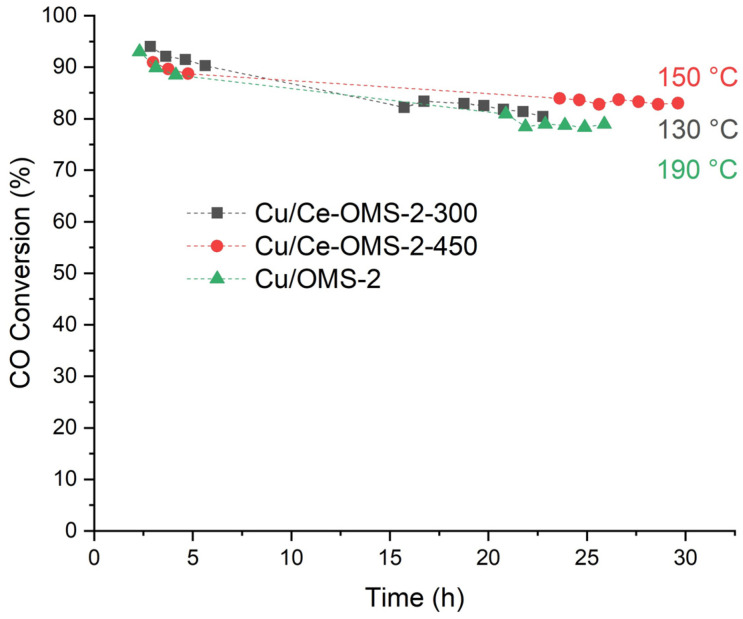
Stability of Cu-containing catalysts in CO oxidation in the presence of water vapor.

The stability of the obtained catalysts was tested in the presence of water vapor (Figure 5). Under “steady-state conditions”, the decrease in the CO conversion is 2.1 and 1.1% % rel. for the Cu/Ce–OMS-2-300 and Cu/Ce–OMS-2-450 samples, respectively. Changing the conditions from light-off mode to those of the stability experiment in the presence of water vapor leads to an increase in the temperature to reach 80% conversion by 16% rel. and 7% rel. for samples Cu/Ce–OMS-2-300 and Cu/Ce–OMS-2-450, respectively, and are 130 °C and 150 °C. The lowest activity in the test is demonstrated by the Cu/OMS-2 sample, and 80% CO conversion under the testing conditions is achieved with an increase in the temperature to up to 190 °C. The changing of the T_80_ values for the Cu/OMS-2 catalyst is 40%, which indicates a significant activity loss.

Thus, the Cu-containing catalysts based on Ce–OMS-2, in which copper was introduced by the method of incipient wetness impregnation, show high activity in the process of catalytic CO oxidation and are stable for at least 25 h in the presence of water vapor. A decrease in the calcination temperature of the Cu/Ce-OMS-2-X system significantly increases the activity of the catalyst in CO oxidation, while maintaining a relatively high stability. In the light-off experiments, the Cu/Ce-OMS-2-300 and Cu/OMS-2 samples are the most active. However, under the conditions of the stability experiments, the latter shows the worst result.

### 3.4. XPS and TEM of Prepared Catalysts

To explain the results of the catalytic studies, the Cu-containing samples were investigated by XPS and HRTEM with EDX mapping. Table 4 shows the positions of the main lines and the quantitative ratios of atoms on the surface according to the XPS results. The increased copper content relative to the one of manganese on the surface of the Cu-Ce/OMS-2-300 sample (Cu/Mn = 0.24) and a decreased copper content for the Cu-Ce/OMS-2-450 (Cu/Mn = 0.12) are observed.

Figure S5 shows the XPS for manganese states. The positions of the Mn 2p, Mn 3s, and Mn 3p lines, i.e., 642.1, 84.3, and 49.7 eV, respectively, indicate the predominant presence of the Mn^4+^ state on the surface of all studied samples. At the same time, the Cu/Ce-OMS-2-450 sample is characterized by the binding energies (BE) of the Mn 3s and Mn 3p levels that are 0.2 and 0.3 eV lower than those of other samples in the series. This indicates a higher content of the reduced state (Mn^3+^). This is also confirmed by the XRD method (Figure 1), where the appearance of reflections related to the Mn_2_O_3_ and Mn_3_O_4_ phases is observed.

Figure S6 shows the Cu 2p and Ce 3d spectra. For all three Cu-containing samples, the photoelectron parameters differ insignificantly: BE(Cu 2p3/2) corresponds to 934.0 ± 0.1 eV, the value of the modified Auger parameter α’(Cu) is 1851.6 ± 0.1 eV, and the F-parameter is 2.3 ± 0.1. These values are the closest to those for CuO, indicating the copper presence on the surface predominantly in the Cu^2+^ state.

Based on the analysis of the Ce 3d line, it was established that on the surface of the cerium-containing samples of the studied series, cerium comprises the Ce^3+^ state by 30 ± 5%, and the rest is presented as the Ce^4+^ state.

The XPS O 1s and C 1s spectra were also recorded (Figure S7). In the case of O 1s, the deconvolution was carried out into three states with binding energies close to 529.5 (OI), 531.4–531.7 (OII), and 533.0–533.2 (OIII) eV. These components can be attributed to the lattice oxygen in the oxide matrix (OI), surface impurity groups in the form of hydroxides, carbonates, or hydrogen carbonates (OII), and adsorbed oxygen-containing molecules H_2_O, CO_2_, or O_2_ (OIII).

In the case of the C 1s regions, the deconvolution was also carried out into three components with binding energies close to 284.7–284.8 (CI), 286.1–286.4 (CII), and 288.1–288.5 (CIII) eV. These components can be attributed to the graphite-like carbon (CI), partially oxidized carbon (CII), and surface carbonate/hydrocarbonate groups (CIIII).

Analyzing the catalytic data and the results of the XPS study, it can be concluded that the CO conversion increases with increasing surface copper content in the samples. A decrease in the copper content in the Cu/OMS-2 and especially Cu/Ce-OMS-2-450 samples leads to a decrease in the catalytic activity with respect to the Cu/Ce-OMS-2-300 sample. Apparently, the increased calcination temperature leads to copper sintering with the formation of larger copper oxide particles. In addition, in the case of the Cu/Ce-OMS-2-450 sample, the increased calcination temperature leads to the formation of individual Mn_2_O_3_ and Mn_3_O_4_ phases and, as a consequence, to an increased surface content of Mn^3+^.

Figure S8 shows the TEM images for the Cu/OMS-2 and Ce-OMS-2 samples. The Cu/OMS-2 sample contains needle-shaped particles with the length varying from 40 nm to 1 μm and the width from 20 to 200 nm. The Ce-OMS-2 sample contains particles of various morphologies. As in the case of the Cu/OMS-2 sample, the Ce-OMS-2 contains needle-shaped particles with the length from 40 to 400 nm and the width from 20 to 200 nm. In addition, the sample contains polycrystalline particles of irregular shape with the size varying from several tens to hundreds of nm. The crystallites that form the agglomerates feature an irregular or needle-like shape, and their size does not exceed 20 nm in the largest dimension.

**Figure 6:**
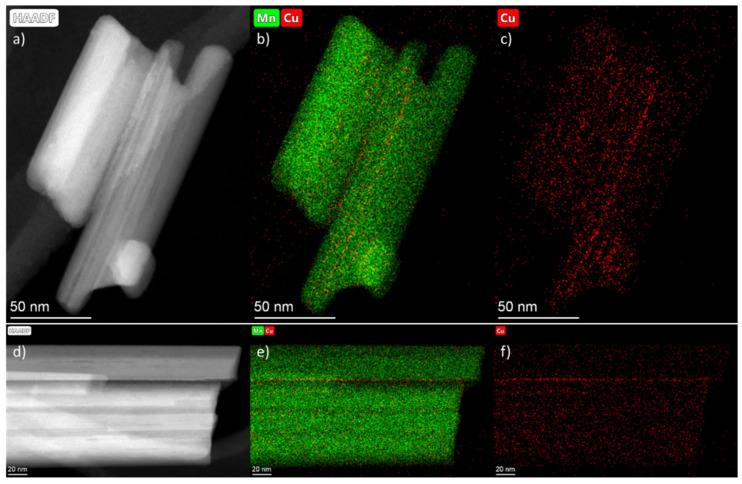
HAADF-STEM images for Cu/OMS-2 sample (**a**,**d**), and EDX mapping (**b**,**c**,**e**,**f**).

To study the copper distribution in the Cu-OMS-2 sample, the EDX mapping was carried out, and the results are shown in Figure 6. The copper-rich areas are present near the surface of the needle-shaped particles as well as along the surface steps. The EDX mapping recorded at lower magnification (Figure S9) allows observing single Cu-rich spherical particles of 15–30 nm in size, which are related to the copper oxide phase also detected by the XRD.

**Figure 7:**
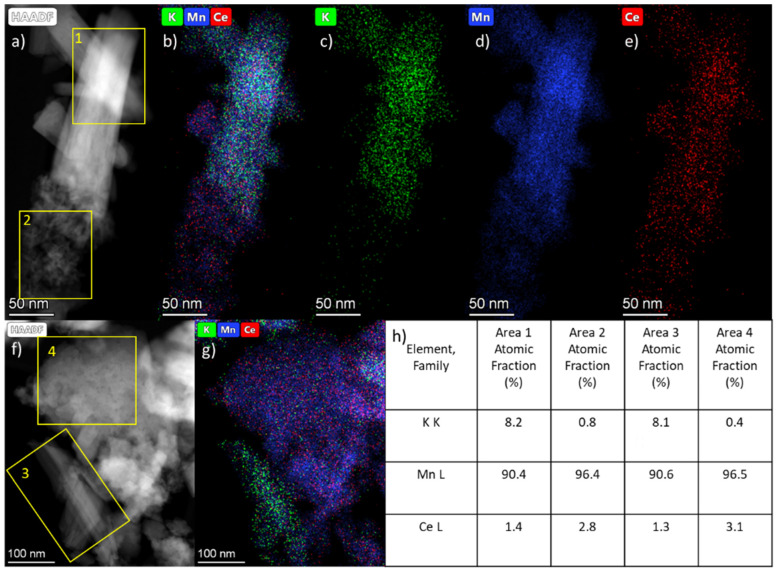
TEM for Ce-OMS-2 sample: (**a**,**f**) HAADF-STEM images, (**b**–**e**,**g**) EDX mapping and (**h**) local elemental composition (excluding oxygen content) in the marked areas.

The distribution of elements in the Ce-OMS-2 sample was studied using the EDX analysis (Figure 7). The agglomerates of polycrystalline particles contain practically no potassium, unlike large needle-shaped crystals. On the other hand, small particles contain approximately twice as much cerium as the needle-shaped crystals. It can be assumed that the polycrystalline particles belong to the pyrolusite phase, since they contain practically no potassium ions. The high concentration of cerium near the pyrolusite phase indicates its direct influence on the phase formation during the hydrothermal treatment process.

**Figure 8:**
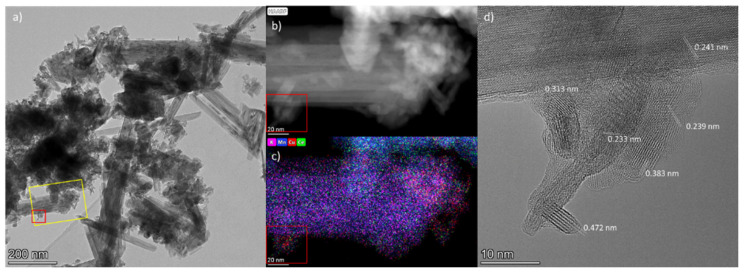
TEM results for Cu-Ce-OMS-2-450 sample: (**a**) TEM image; (**b**,**c**) HAADF-STEM image of yellow-marked area in (**a**), and EDX mapping; (**d**) HRTEM image of red-marked area in images (**a**–**c**) with interplanar distances.

**Figure 9:**
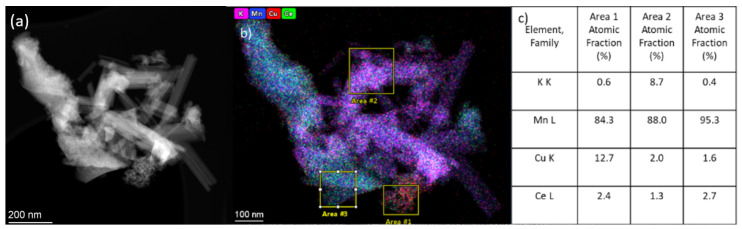
TEM results for Cu-Ce-OMS-2-300 sample: (**a**,**b**) HAADF-STEM image and EDX mapping; (**c**) local elemental composition excluding oxygen in marked areas in (**b**).

Figure 8 and Figure 9, respectively, show the TEM data for the Cu/Ce-OMS-2-450 and Cu/Ce-OMS-300 samples. In these samples, as well as in the Ce-OMS-2, the particles of various morphologies, individual needle-shaped crystals, and polycrystalline agglomerates are observed. According to the EDX analysis, heterogeneity in the local distribution of elements takes place. In addition to the peculiarities of the distribution of potassium and cerium, as observed for the Ce-OMS-2 sample, there are heterogeneities in the Cu distribution. Thus, for some polycrystalline agglomerates, an increased copper content is observed compared to other areas of the sample (Figure 8 and Figure 9b,c, area 1). The HRTEM images of such areas are shown in Figure 8d.

The analysis of the HRTEM images reveals interplanar distances in the Cu/Ce-OMS-2-450 sample (0.472 nm, 0.383 nm), which can be attributed to Mn_2_O_3_. The distance of 0.233 nm in the center of the particle agglomerate in Figure 8d can be attributed to CuO due to the slightly increased copper concentration in this region in the EDX map (d_200_ CuO = 0.231 nm).

### 3.5. Discussion

There has been a long-standing debate in the literature regarding the mechanism of action of Cu-MnO_x_ catalytic systems for CO oxidation. Some studies [51,52] report the formation of spinel structures of the CuMnO_x_ type on the surface as an active phase in CO oxidation. However, other studies (e.g., [38]) report the importance of the interphase boundary between a mixture of CuO and Mn_2_O_3_ oxides for the formation of the redox pair Cu^2+^ + Mn^3+^ = Cu^+^ + Mn^4+^. In Ref. [5], for systems like those considered in the present work, the authors reported the importance of the formation of the Cu^2+^–O–Mn^4+^ interface for the CO oxidation process. Oxygen species in this group feature higher mobility compared to the individual copper and manganese oxides, which provides these systems with activity in low-temperature CO oxidation through the formation of oxygen vacancies. Based on the results obtained, we assume the formation of such an interface on the surface of the Cu/OMS-2 sample.

On the other hand, the study of the reaction in the presence of water vapor shows an increased stability of the Ce-containing systems compared to the Cu/OMS-2 sample. It is known from the literature [53] that CeO_2_ is capable of being an oxygen donor for manganese oxide at low temperatures. Also, in Ref. [10], the redox pair Mn^3+^ + Ce^4+^ ↔ Mn ^4+^ + Ce^3+^ is mentioned. Due to the effects described above, the activity of the Cu^2+^–O–Mn^4+^ interface increases due to the abundant presence of surface oxygen vacancies near Ce^3+^ increasing the activation of molecular oxygen. This facilitates the Mn^3+^ reoxidation under wet conditions. Based on the stability test results in the process of low-temperature CO oxidation in the presence of water vapor, it can be concluded that there is a synergistic interaction of Cu-, Mn- and Ce-containing sites in the catalysts of the Cu/Ce-OMS-2-X composition.

There are a lot of publications concerning the Ce introduction into the OMS-2 structure through one-pot hydrothermal synthesis [15,20,54]. However, most publications do not provide comprehensive evidence of the formation of the OMS-2 structure during the hydrothermal treatment in the presence of cerium ions. In our work, it is shown for the first time that the introduction of cerium nitrate as a precursor by one-pot hydrothermal synthesis with Mn salts leads to the appearance of an additional pyrolusite phase. In Ref. [55], the growth of α-MnO_2_ rods was shown to occur through recrystallization with the completion of the particles with the [MnO_6_] octahedra. The results obtained in the current work allow concluding that the Cu introduction into the Ce-OMS-2 system allows us to reconstruct the cryptomelane structure at least at the particle interfaces. It seems that close contact between the pyrolusite and cryptomelane phases allows the completion of the structure with the 2 × 2 tunnels at the interface.

## 4. Conclusions

In this work, the OMS-2-based catalysts modified with cerium by the hydrothermal treatment method and the Cu-containing species were synthesized. The introduction of cerium nitrate at the stage of the co-precipitation of MnO_x_ precursors before the hydrothermal treatment prevented the complete formation of the 2 × 2 tunnels of the OMS-2 with the formation of the pyrolusite phase. The Cu introduction by incipient wetness impregnation into such a system is accompanied by a partial reconstruction of the cryptomelane structure at the phase boundary by incorporating Cu^2+^ into the cation vacancies of the [MnO_6_] octahedra. Increasing the calcination temperature to up to 450 °C led to the reverse destruction of the 2 × 2 tunnel structure with a decrease in the dispersion of copper oxide and the reduction in manganese oxides to Mn_2_O_3_ and Mn_3_O_4_.

The catalytic activity of the OMS-2 systems in the low-temperature CO oxidation increased with the introduction of cerium and copper into the system. In the light-off mode, the Cu/Ce-OMS-2-300 and Cu/OMS-2 samples turned out to be the most active catalysts. The catalytic activity under conditions without water vapor was determined by the presence of the Cu^2+^–O–Mn^4+^ surface ensembles with a high mobility of the oxygen atom, which took part in the CO oxidation.

However, when water vapor was added, the presence of additional sites was necessary to facilitate the activation of molecular oxygen. Such sites were Ce^3+^ cations providing sufficient reoxidation of the surfaces of the Cu/Ce-OMS-2-300 and Cu/Ce-OMS-2-450 catalysts. The results obtained in this work deepen the understanding of the mechanism of redox processes occurring on the MnO_2_-based catalysts modified by Cu- and Ce-containing species.

## Figures and Tables

**Table 1:** Elemental composition and textural characteristics of prepared catalysts.

Sample	T _calc._, °C	XRF			Textural Characteristics			
		Ce/∑Me	Cu/∑Me	K/∑Me	S_BET_ m^2^/g	V_pore._ cm^3^/g	d_meso,_ nm	d_av_, nm
OMS-2	450	–	–	0.120	15.9	0.07	21.4	378
Ce-OMS-2	450	0.016	–	0.038	46.5	0.17	14.9	129
Cu/OMS-2	450	–	0.049	0.122	16.7	0.08	22.3	359
Cu/Ce-OMS-2-450	450	0.015	0.051	0.035	37.3	0.15	16.8	161
Cu/Ce-OMS-2-300	300	0.014	0.053	0.032	40.5	0.18	15.0	148

**Table 2:** Unit cells and phase composition of prepared catalysts.

Sample	Phase	Phase Content, wt. %	Lattice Parameter, A	CSR, nm
Cu/Ce-OMS-2-300	OMS-2 (JCPDS 29-1020)	30	a: 9.7369 c: 2.8762	18
	β-MnO_2_ (JCPDS 24-0735)	70	a: 4.4044 c: 2.8621	16
Ce-OMS-2	OMS-2 (JCPDS 29-1020)	30	a: 9.7403 c: 2.8682	20
	β-MnO_2_ (JCPDS 24-0735)	70	a: 4.3922 c: 2.8677	13
Cu/OMS-2	OMS-2 (JCPDS 29-1020)	100	a: 9.8153 c: 2.8553	30
Cu/Ce-OMS-2-450	OMS-2 (JCPDS 29-1020)	30	a: 9.7514 c: 2.8713	20
	β-MnO_2_ (JCPDS 24-0735)	70	a: 4.3969 c: 2.8717	13

**Table 3:** H_2_ consumption for prepared catalysts.

Sample	H_2_ Consumption, mmol/g
OMS-2	7.98
Cu/OMS-2	8.14
Ce–OMS-2	5.97
Cu/Ce–OMS-2-450	6.79
Cu/Ce–OMS-2-300	8.07

**Table 4:** XPS lines and chemical composition of surface for prepared catalysts.

Sample	Mn 2p	Mn 3s	Mn 3p	Cu 2p	α’(Cu)	O1s	Cu/Mn	Ce/Mn	K/Mn
OMS-2	642.1	84.3	49.7			529.5			0.060
Ce/OMS-2	642.1	84.3	49.7			529.5		0.045	0.014
Cu/OMS-2	642.1	84.3	49.7	934.1	1851.7	529.5	0.19		0.068
Cu-Ce/OMS-2-300	642.1	84.3	49.7	934.0	1851.6	529.5	0.24	0.041	0.014
Cu-Ce/OMS-2-450	642.0	84.1	49.4	934.0	1851.7	529.5	0.12	0.044	0.013

## Data Availability

Dataset available on request from the authors.

## Supplementary Materials

The following supporting information can be downloaded at: https://www.mdpi.com/article/10.3390/nano15030166/s1. Figure S1. Textural characteristics of prepared catalysts: Isotherms of adsorp-tion-desorption (a) and pore size distribution (b). Figure S2. Morphology images obtained by SEM: OMS-2 (a), Ce-OMS-2 (b), Cu/OMS-2 (c), Cu/Ce-OMS-2-450 (d) and Cu/Ce-OMS-2-300 (e). Figure S3. Cryptomelane (a) and pyrolusite (b) unit cells. Figure S4. XRD pattern modeling for: (a) Ce-OMS-2, (b) Cu/OMS-2, (c) Cu/Ce-OMS-2-450, (d) Cu/Ce-OMS-2-300 samples made with POWDER CELL 2.4 full-profile analysis program. Figure S5. Mn 2p, Mn 3s and Mn 3p XP spectra. Figure S6. Cu 2p and Ce 3d XP spectra. Figure S7. O 1s and C 1s XP spectra. Figure S8. TEM images for Cu/OMS-2 (a,c) and Ce-OMS-2 (b,d) samples: (a,b) particle morphology; (c,d) HRTEM images. Figure S9. HAADF-STEM images for Cu-OMS-2 samples (a) and EDX mapping (b). Table S1. Comparison of CO oxidation performance for the prepared sample and state-of-the-art catalysts reported in literature. References [56,57,58,59,60] are cited in the supplementary materials.
