# Supplementary material for: Unravelling the Cu and Ce Effects in MnO2-Based Catalysts for Low-Temperature CO Oxidation
Source: Nanomaterials (Basel). 2025 Jan 22;15(3):166. doi: 10.3390/nano15030166 (PMC11819697; doi:10.3390/nano15030166)
Supplement: Supplementary file 1 [file nanomaterials-15-00166-s001.zip › nanomaterials-3423677-supplementary.pdf]

nanomaterials

MDPI

Supporting Information

Unravelling the Cu and Ce Effects in MnO_2-Based Catalysts for Low-Temperature CO Oxidation

Egor D. Blinov [1], Ekaterina V. Kulchakovskaya [1], Nikolai A. Sokovikov [2], Valery A. Svetlichnyi [3], Sergei A. Kulinich [4,*] and Olga V. Vodyankina [1,*]

[1] Department of Physical and Colloid Chemistry, Faculty of Chemistry, Tomsk State University, Tomsk 634050, Russia; egir.blinov@gmail.com (E.D.B.); ekaterina.krv@gmail.com (E.V.K.)
[2] Department of Physical Chemistry, Faculty of Natural Science, Novosibirsk State University, Novosibirsk 630090, Russia; n.sokovikov@g.nsu.ru
[3] Laboratory of Advanced Materials and Technology, Tomsk State University, Tomsk 634050, Russia; v_svetlichnyi@bk.ru
[4] Research Institute of Science and Technology, Tokai University, Hiratsuka, Kanagawa 259-1292, Japan
* Correspondence: skulinich@tokai-u.jp (S.A.K.); vodyankina_o@mail.ru (O.V.V.)

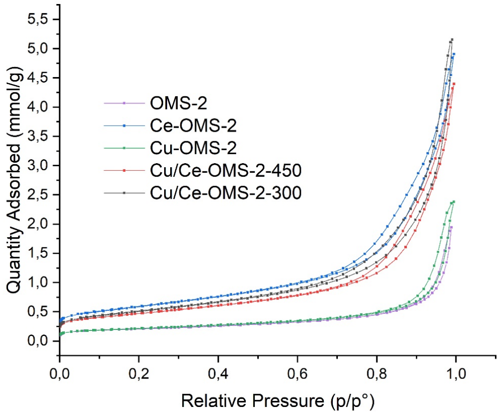

(a)

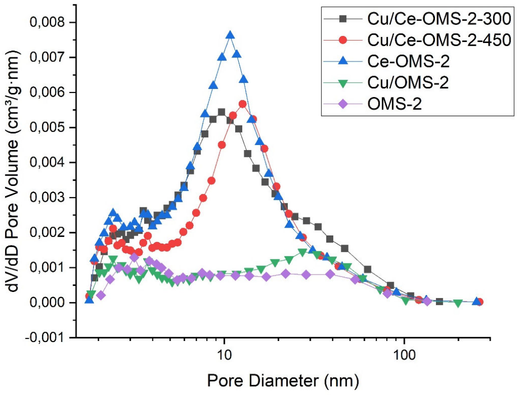

(b)

Figure S1. Textural characteristics of prepared catalysts: Isotherms of adsorption-desorption **(a)** and pore size distribution **(b)**.

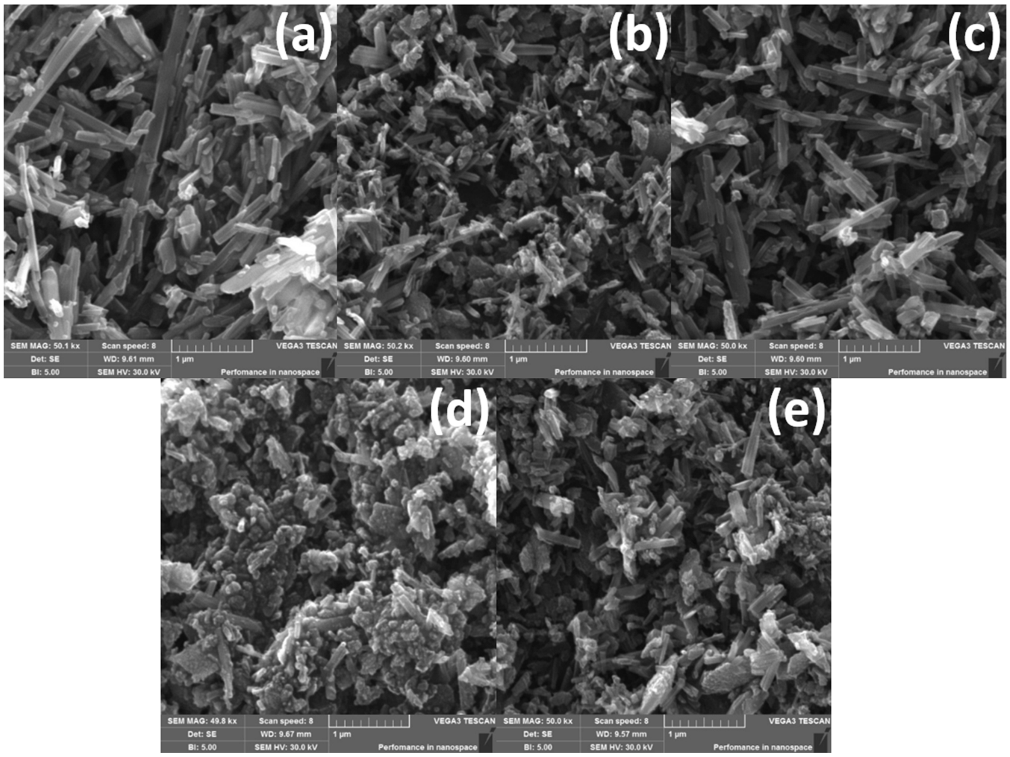

Figure S2. Morphology images obtained by SEM: OMS-2 **(a)**, Ce-OMS-2 **(b)**, Cu/OMS-2 **(c)**, Cu/Ce-OMS-2-450 **(d)** and Cu/Ce-OMS-2-300 **(e)**.

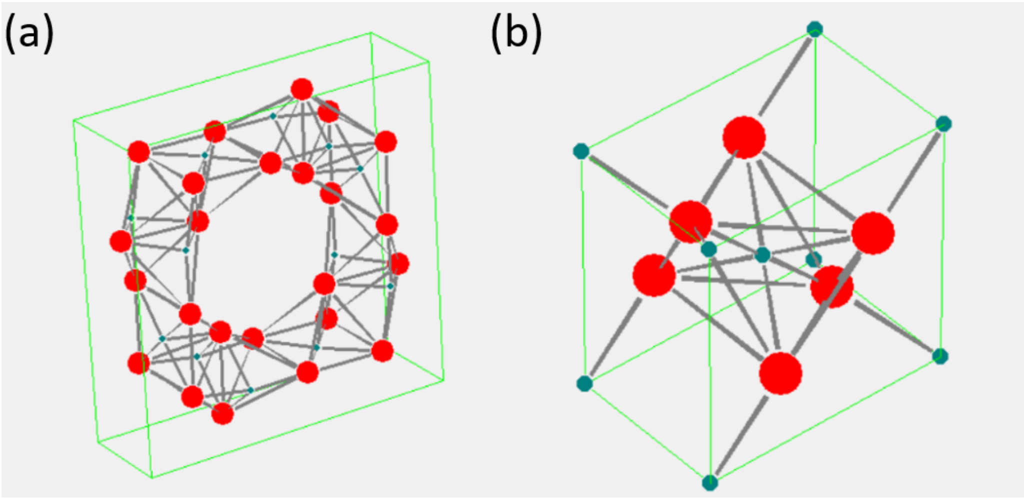

Figure S3. Cryptomelane **(a)** and pyrolusite **(b)** unit cells.

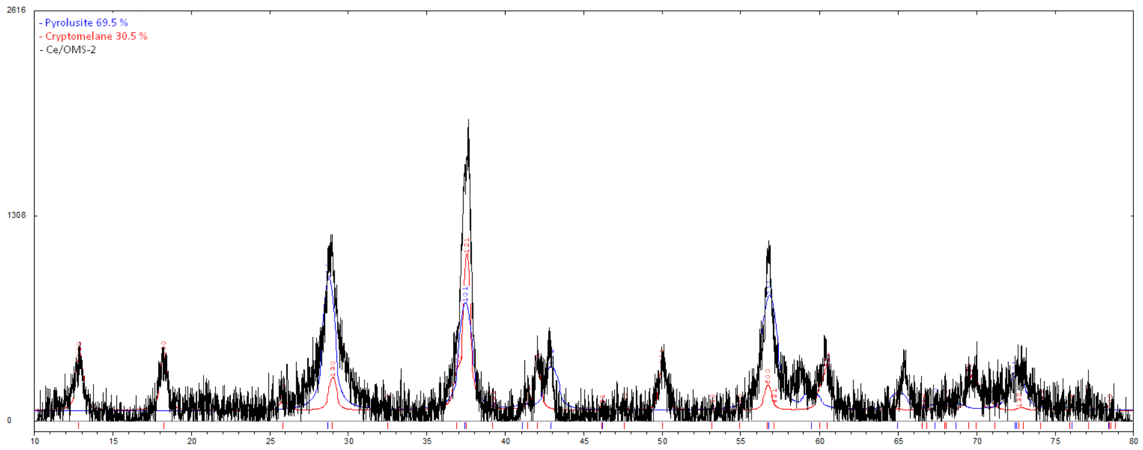

- Pyrolusite 69.5 %
- Cryptomelane 30.5 %
- Ce/OMS-2

(a)

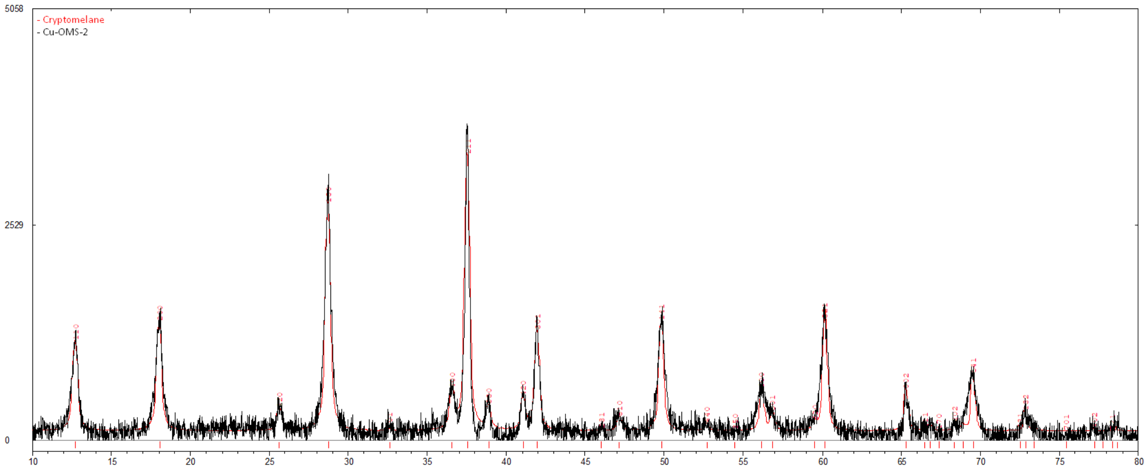

- Cryptomelane
- Cu-OMS-2

(b)

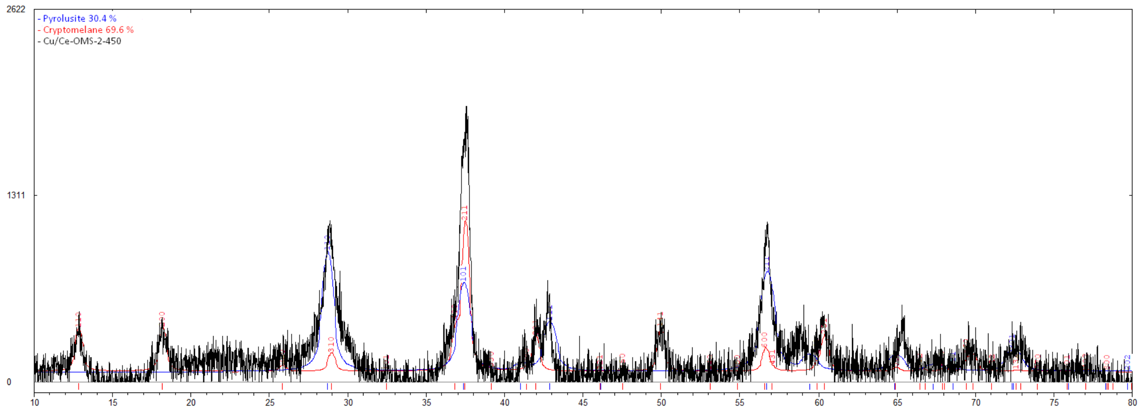

- Pyrolusite 30.4 %
- Cryptomelane 69.6 %
- Cu/Ce-OMS-2-450

(c)

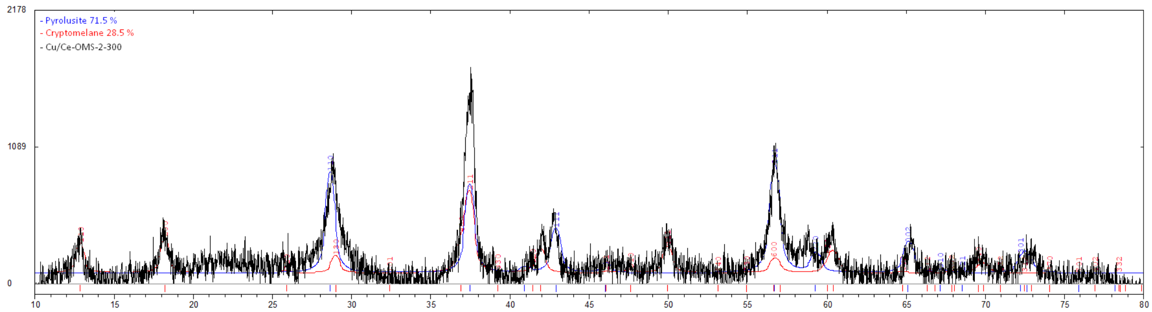

(d)

Figure S4. XRD pattern modeling for: **(a)** Ce-OMS-2, **(b)** Cu/OMS-2, **(c)** Cu/Ce-OMS-2-450, **(d)** Cu/Ce-OMS-2-300 samples made with POWDER CELL 2.4 full-profile analysis program.

Table S1. Comparison of CO oxidation performance for the prepared sample and state-of-the-art catalysts reported in literature.

**Catalyst**	**Preparation method**	**Reaction conditions**	**Flow rate / SV / GHSV**	**T_{90} (°C)**	**Year**	**Ref**
4% Cu/Ce-OMS-2-300	Hydrothermal+ Wet impregnation	1% CO, 8% O_2 in He	90000 mL*g^{-1}*h^{-1}	115	2023	Present work
Ce-Mn mixed oxide	Citrate-hydrothermal	0,15% CO, 20% O_2 in N_2	30000 mL*g^{-1}*h^{-1}	110	2023	[1]
CuO-Mn_2O_3	Hydrolysis-hydrothermal	1% CO, 1% O_2 in N_2	17140 mL*g^{-1}*h^{-1}	103	2022	[2]
10% CuO_x/CeO_2	Hydrothermal+ Wet impregnation	1% CO, 20% O_2 in He	76000 mL*g^{-1}*h^{-1}	173	2022	[3]
5% CuO/Al_2O_3	Wet impregnation	2% CO, 20.8% O_2 in He	30000 mL*g^{-1}*h^{-1}	218	2023	[4]
1.5% Pt/TiO_2	Photochemical deposition	1% CO, 1% O_2 in Ar	30000 mL*g^{-1}*h^{-1}	112	2023	[5]
1% Au/NiO	Hydrothermal+ Impregnation with colloidal solution	1% CO, 20% O_2 in He	60000 mL*g^{-1}*h^{-1}	158	2024	[6]
Au/Fe_2O_3	Salt-assisted ultrasonic spray pyrolysis + Deposition-precipitation	2% CO, 10% O_2 in Ar	120000 mL*g^{-1}*h^{-1}	53	2022	[7]
0.45% Pt/g-C_3N_4	Ethylene glycol reduction	1.05% CO, 20% O_2 in Ar	30000 mL*g^{-1}*h^{-1}	184	2023	[8]
1% $Pt/CeZrO_x$	Wet impregnation + atom trapping	0.5% CO, 10% O_2 in Ar	150000 mL*g^{-1}*h^{-1}	67	2024	[9]
1% Pt/TiO_2	Sol-gel	1% CO, 1% O_2 in N_2	150000 mL*g^{-1}*h^{-1}	110	2022	[10]

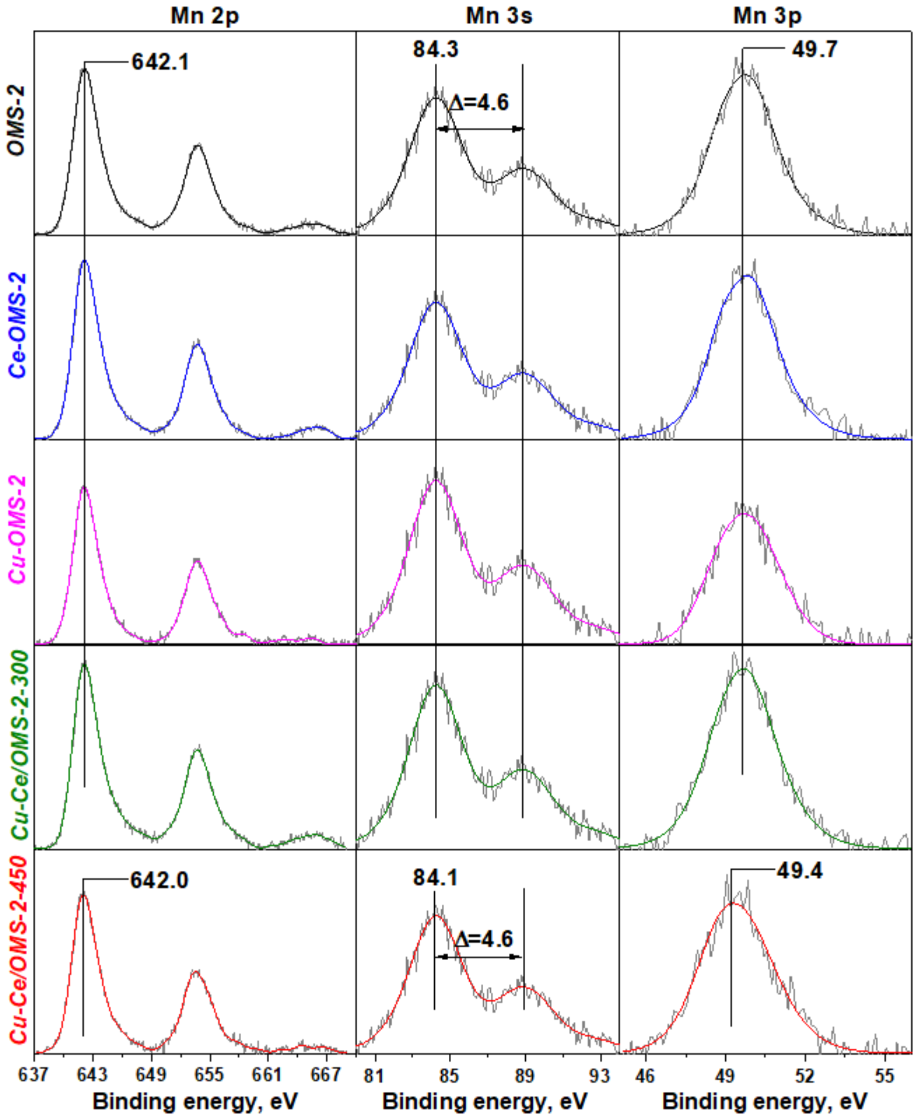

Figure S5. Mn 2p, Mn 3s and Mn 3p XP spectra.

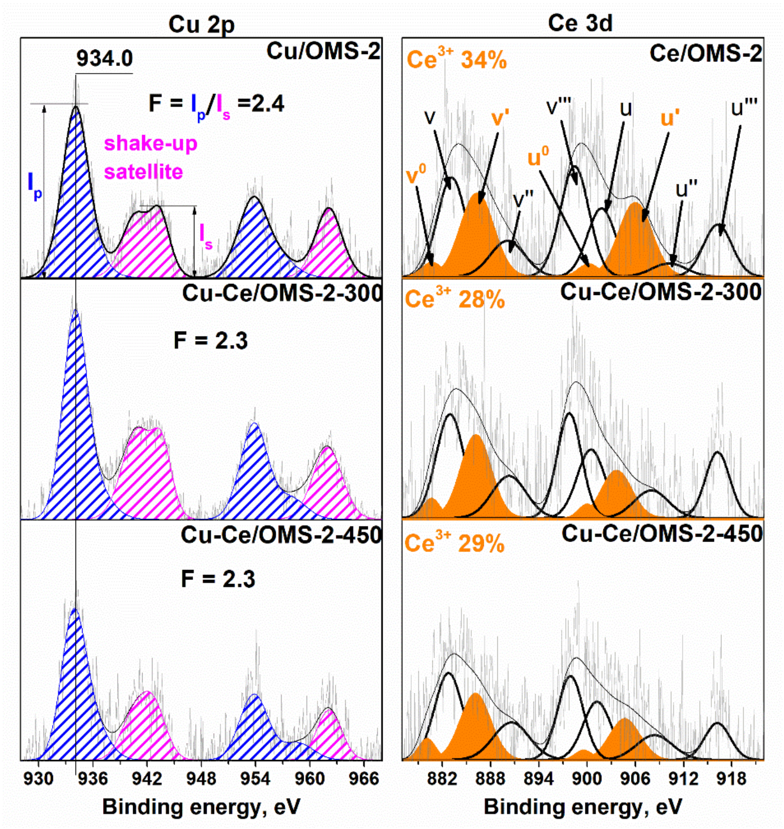

Figure S6. Cu 2p and Ce 3d XP spectra.

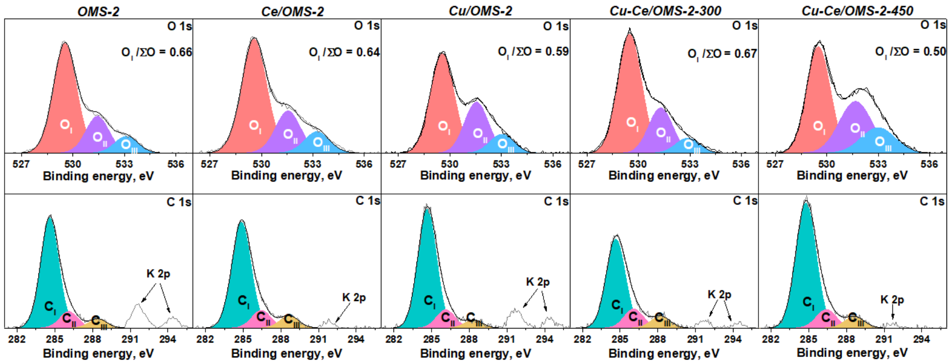

.

Figure S7. O 1s and C 1s XP spectra.

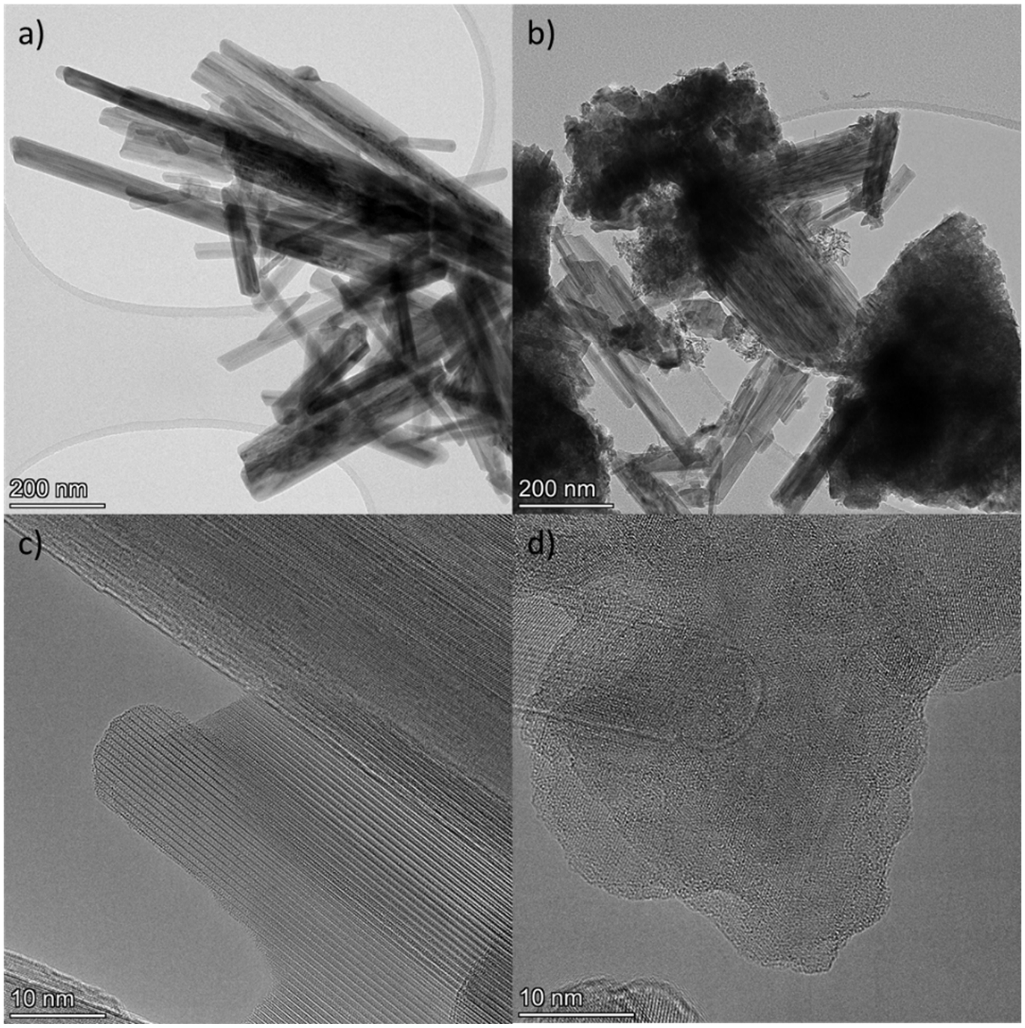

Figure S8. TEM images for Cu/OMS-2 **(a,c)** and Ce-OMS-2 **(b,d)** samples: a-b) particle morphology; c-d) HRTEM images.

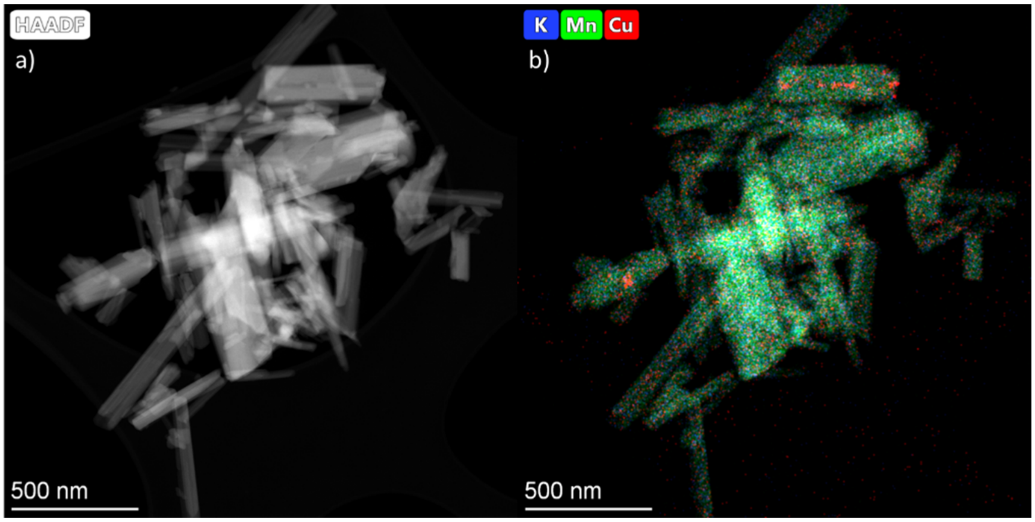

Figure S9. HAADF-STEM images for Cu-OMS-2 samples **(a)** and EDX mapping **(b)**.

References

1. Ye, Z.; Liu, Y.; Nikiforov, A.; Ji, J.; Zhao, B.; Wang, J. The research on CO oxidation over Ce–Mn oxides: The preparation method effects and oxidation mechanism. *Chemosphere* **2023**, *336*, 139130.
2. Wang, J.; Chen, J.; Peng, L.; Zhang, H.; Jiang, Z.; Xiong, K.; Yang, N. On the CuO-Mn_2O_3 oxide-pair in $CuMnO_x$ multi-oxide complexes: Structural and catalytic studies. *Appl. Surf. Sci.* **2022**, *575*, 151733.
3. Ahasan, M.R.; Wang, Y.; Wang, R. In situ DRIFTS and CO-TPD studies of CeO_2 and SiO_2 supported CuOx catalysts for CO oxidation. *Mol. Catal.* **2022**, *518*, 112085.
4. Lv, J.; Chen, C.; Guo, X.; Ding, W.; Yang, W. Crystal facet effect of γ-Al_2O_3 on catalytic property of CuO/γ-Al_2O_3 for CO oxidation. *Mol. Catal.* 2023, 547, 113405.
5. He, W.; Zhang, X.; Zheng, K.; Wu, C.; Pan, Y.; Li, H.; Wei, S. Structural evolution of anatase-supported platinum nanoclusters into a platinum-titanium intermetallic containing platinum single atoms for enhanced catalytic CO oxidation. *Angew. Chem. Int. Ed.* **2023**, *62*, e202213365.
6. Barkaoui, S.; Wang, Y.; Zhang, Y.; Gu, X.; Li, Z.; Wang, B.; Zhao, Z. Critical role of NiO support morphology for high activity of Au/NiO nanocatalysts in CO oxidation. *iScience* **2024**, *27*, 110255.
7. Sarkodie, B.; Shen, B.; Asinyo, B.; Hu, Y.; Jiang, J.; Li, C. Highly efficient Au/Fe_2O_3 for CO oxidation: the vital role of spongy Fe_2O_3 toward high catalytic activity and stability. *J. Colloid Interface Sci.* **2022**, 608, 2181-2191.
8. Bi, F.; Ma, S.; Gao, B.; Yang, Y.; Wang, L.; Fei, F.; Zhang, X. Non-oxide supported Pt-metal-group catalysts for efficiently CO and toluene co-oxidation: Difference in water resistance and degradation intermediates. *Fuel*. **2023**, 344, 128147
9. Liu, Z.; Liu, K.; Yang, X.; Chen, X.; Shen, X.; Li, Y.; Zhang, Y. In-situ formed stable Pt nanoclusters on ceria-zirconia solid solutions induced by hydrothermal aging for efficient low-temperature CO oxidation. *J. Chem. Eng.* **2024**, 498, 155427.
10. Camposeco, R.; Torres, A.E.; Zanella, R. Influence of the preparation method of Au, Pd, Pt, and Rh/TiO_2 nanostructures and their catalytic activity on the CO oxidation at low temperature. *Top. Catal.* **2022**, 65, 798-816
